# Identification of MAD2L1 as a Potential Biomarker in Hepatocellular Carcinoma via Comprehensive Bioinformatics Analysis

**DOI:** 10.1155/2022/9868022

**Published:** 2022-01-28

**Authors:** Qian Chen, Sibo Yang, Yewei Zhang, Bo Li, Huanming Xu, Shi Zuo

**Affiliations:** ^1^Department of Clinical Medicine, Guizhou Medical University, Guiyang, China; ^2^The First Affiliated Hospital of Guangzhou Medical University, Guangzhou, China; ^3^Department of Hepatobiliary Surgery, The Affiliated Hospital of Guizhou Medical University, Guiyang, China

## Abstract

**Background:**

Hepatocellular carcinoma (HCC) is widely acknowledged as a malignant tumor with rapid progression, high recurrence rate, and poor prognosis. At present, there is a paucity of reliable biomarkers at the clinical level to guide the management of HCC and improve patient outcomes. Our research is aimed at assessing the prognostic value of MAD2L1 in HCC.

**Methods:**

Four datasets, GSE121248, GSE101685, GSE85598, and GSE62232, were selected from the GEO database to analyze differentially expressed genes (DEGs) between HCC and normal liver tissues. After functional analysis, we constructed a protein-protein interaction network (PPI) for DEGs and identified core genes in this network with high connectivity with other genes. We assessed the relationship between core genes and the pathogenesis and prognosis of HCC. Finally, we explored the gene regulatory signaling mechanisms involved in HCC pathogenesis.

**Results:**

145 DEGs were screened from the intersection of the four GEO datasets. MAD2L1 was associated with most genes according to the PPI network and was selected as a candidate gene for further study. Survival analysis suggested that high MAD2L1 expression in HCC correlated with a worse prognosis. In addition, real-time quantitative PCR (RT-qPCR), western blot (WB), and immunohistochemistry (IHC) findings suggested that the expression of MAD2L1 was abnormally increased in HCC tissues and cells compared to paraneoplastic tissues and normal hepatocytes.

**Conclusion:**

We found that high MAD2L1 expression in HCC was significantly associated with overall patient survival and clinical features. We also explored the potential biological properties of this gene.

## 1. Introduction

According to the latest World Health Organization estimates, hepatocellular carcinoma (HCC) is one of the most common malignancies in the world [1, 2], with high heterogeneity [3], recurrence, and metastatic rates [4] and a poor long-term prognosis. Although surgical resection is the mainstay of treatment for early-stage HCC [5], most HCC patients are already at an advanced stage at diagnosis. Accordingly, it is essential to elucidate the molecular mechanisms of HCC [6, 7] and identify effective molecular targets [8, 9] to improve patient survival and quality of life [10].

Mitotic arrest deficient 2-like protein 1 (MAD2L1) has been recognized as an important member of the MAD2 family [11]. MAD2L1 is a protein-coding gene [12] and a mitotic spindle assembly checkpoint component that prevents anaphase onset until all chromosomes are correctly aligned at the metaphase plate [13]. Until recently, the correlation between the overexpression of MAD2L1 in tumors and its prognostic value has been demonstrated [14]. In this regard, MAD2L1 has been documented to be overexpressed in lung adenocarcinoma cells and can promote proliferation and inhibit apoptosis [15]. However, there is limited evidence regarding the association between MAD2L1 and tumors, and the role of MAD2L1 in HCC remains unclear.

Gene Expression Omnibus (GEO) is a comprehensive online cancer research database that provides high-throughput gene expression data submitted by research institutions from all over the world [16]. In the current study, we selected several mRNA microarray datasets (GSE121248, GSE101685, GSE85598, and GSE62232) from the GEO database to screen for differentially expressed genes associated with HCC occurrence and development. We then performed a series of bioinformatics analyses such as the protein-protein interaction (PPI) network construction, Kaplan-Meier survival analysis, and functional analysis to identify key genes that may regulate HCC progression [17]. In addition, microarray tissue samples were utilized to investigate the potential clinical relevance of key genes. The findings of this study offer valuable insights into the quest for new markers and drug candidate genes for targeted therapy in HCC patients.

## 2. Materials and Methods

### 2.1. Data Acquisition

Four HCC datasets (GSE121248, GSE101685, GSE84598, and GSE62232) were downloaded from the NCBI GEO database. Dataset GSE121248 consisted of 70 HCC and 37 paraneoplastic tissue samples, GSE101685 contained 25 HCC and 25 paraneoplastic tissue samples, GSE62232 included 81 HCC and 9 paraneoplastic tissue samples, and GSE84598 contained 22 HCC and 22 paraneoplastic tissue samples. DEGs were screened from the above 4 datasets. RNA sequencing data and survival prognosis data for the hub genes were obtained from The Cancer Genome Atlas (TCGA) database.

### 2.2. Identification of DEGs

GEO2R is an online tool used to conduct differential gene expression analysis on GEO data. DEGs in HCC and paracancer samples identified by GEO2R were downloaded from the GEO database with the cDNA expression profiles. The log-fold change (FC) in expression and adjusted *P* values (adj.*P*) were determined. Genes that met the cutoff criteria of adj.*P* < 0.05 and ∣logFC | >1.0 were regarded as DEGs [16]. Genes from the four datasets were intersected using the Venn diagram network tool. Volcano plots of DEGs were generated by visual hierarchical clustering analysis.

### 2.3. Functional Enrichment Analysis of DEGs

To reveal the functions of DEGs, we used the Enrichr database for GO annotation and KEGG pathway enrichment analysis [18]. The GO terminology consists of three components: biological process (BP), cellular component (CC), and molecular function (MF). An adj.*P* value < 0.05 was statistically significant.

### 2.4. Hub Gene Analysis

To query the potential correlation between these DEGs, we evaluated them using the STRING web tool [19]. Furthermore, modules of the PPI network (cutoff values were set to degree = 2, node score = 0.2, *k*‐core = 2, and maximum depth = 100) were explored using the Molecular Complex Detection (MCODE) plugin from Cytoscape.

### 2.5. Gene Set Enrichment Analysis

All gene expression data of TCGA-HCC were downloaded from the UCSC Xena platform. The HCC patients were divided into high-expression and low-expression groups, according to the median value of MAD2L1 expression [20]. Gene set enrichment analysis (GSEA) [21] evaluated expression differences in gene sets between the two groups to validate GO and KEGG analysis results. An adj.*P* value < 0.05 was statistically significant.

### 2.6. Survival Analysis

The Kaplan-Meier plotter is an analysis tool built by Oncomir that can be used to analyze the overall survival of various tumors with genes, featuring a total of 54675 genes in a sample of 10461 patients. In this study, Kaplan-Meier analysis was conducted to examine the correlation between MAD2L1 expression and OS and RFS in HCC patients. The log-rank test determined whether the two survival curves were statistically significantly different. *P* value < 0.01 was statistically significant.

### 2.7. The Quantitative Real-Time Polymerase Chain Reaction of Cell Lines

Cells including LO2, human normal liver cell line, and HCC cell lines HCCLM3, MHCC-97H, Huh7, HepG2, and Hep3B were obtained from the Chinese Cell Bank (Shanghai, China) and cultured with Dulbecco's modified Eagle medium supplemented with 10% fetal bovine serum. All cells were incubated at 37°C in a 5% CO2 incubator.

The TRIzol reagent (Invitrogen, Thermo Fisher Scientific, Shanghai, China) was applied to extract total RNA from cell lines according to the manufacturer's instructions. Reverse transcription into cDNA was conducted using the Transcriptor First Strand cDNA Synthesis Kit. The cDNA strand was analyzed by qRT-PCR using the SYBR PCR kit. The expression of MAD2L1 was computed by the 2^(−ΔΔCt)^ method using GAPDH as an internal reference. The qRT-PCR primers used in the present study were as follows: MAD2L1 forward primer, 5′-GTTCTTCTCATTCGGCATCAACA-3′; MAD2L1 reverse primer, 3′-GAGTCCGTATTTCTGCACTCG-5′; GAPDH forward primer, 5′-CACCATGAAGATCAAGATCATTGC-3′; and GAPDH reverse primer, 3′-GGCCGGACTCATCGTACTCCTGC-5′.

### 2.8. Western Blot

Cells were lysed in RIPA buffer containing 1% protease inhibitor PMSF. Centrifugation of the upper supernatant was performed, and the protein levels were determined by the BCA protein assay. Total proteins were separated on 12.5% SDS gels at 80 V for 30 minutes, followed by 120 V for 60 minutes. The protein was transferred onto PVDF membranes at 350 mA for 1 hour. Then, the protein blots were incubated with primary antibodies consisting of MAD2L1 (10337-1-AP, PTG, 1 : 1000) and *β*-tubulin (AP0064, Bioworld, 1 : 5000) for 14-18 hours. After the blots were incubated with secondary antibodies, bands were detected by enhanced chemiluminescence.

### 2.9. Evaluation of IHC Results

Tissue arrays were purchased from Shanghai Outdo Biotechnology Co. (Shanghai Outdo, Shanghai, China) and applied to estimate the expression of MAD2L1 protein.

The IHC results based on the staining intensity and staining area of the tissue microarrays were scored by two experienced pathologists from the Department of Pathology at the Affiliated Hospital of Guizhou Medical University [22]. The staining area was scored as follows: 0, <5%; 1, 6-25%; 2, 26-50%; 3, 51-75%; and 4, >75%. The staining intensity was scored as follows: 0, none; 1, mild; 2, moderate; and 3, strong. The score for each segment was equal to the product of their staining intensity and staining area and was categorized as negative if the final score was <6 and positive if the final score was ≥6.

### 2.10. Statistical Analysis

SPSS 22.0 (SPSS, IL, USA) was used for statistical analysis. Student's two-tailed *t*-test was used to assess statistical significance of differences between two groups and one-way ANOVA among multiple groups. The Wilcoxon rank-sum test analyzed the skewed data. Spearman's rank correlation test was conducted to assess the correlation between gene expression levels. The chi-square test evaluated correlations between gene expression and clinicopathological characteristics. Survival analysis was conducted by the Kaplan-Meier method and log-rank test. Univariate and multivariate survival analyses were calculated using Cox proportional regression models. A *P* value < 0.05 indicated statistical significance (^∗^*P* < 0.05, ^∗∗^*P* < 0.01, and ^∗∗∗^*P* < 0.001).

## 3. Results

### 3.1. Identification of 145 DEGs in HCC Compared to Normal Liver Tissue

**Figure 1:**
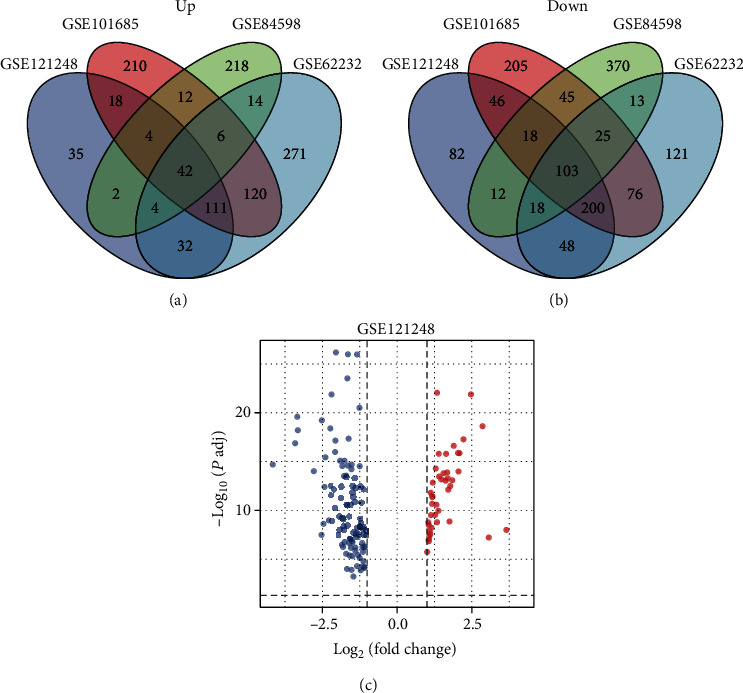
Identification of common DEGs from GSE121248, GSE101685, GSE85598, and GSE62232 datasets: (a) the 42 upregulated DEGs; (b) The 103 downregulated DEGs; (c) volcano plot of the 145 DEGs (red, upregulation; blue, downregulation).

Four datasets (GSE121248, GSE101685, GSE84598, and GSE62232) were obtained from the GEO database to analyze DEGs between HCC and paraneoplastic tissues. 775 DEGs (248 upregulated and 527 downregulated genes) were identified in the GSE121248 dataset. 1241 DEGs (523 upregulated and 718 downregulated genes) were obtained from dataset GSE101685. Moreover, for the GSE84598 dataset, 906 DEGs (302 upregulated and 604 downregulated genes) were identified. Finally, 1204 DEGs (600 upregulated and 604 downregulated genes) were screened from GSE62232. Remarkably, 42 DEGs were significantly upregulated (Figure 1(a)), and 103 DEGs were downregulated (Figure 1(b)) in HCC tissues compared with paracancerous tissues (Supplementary Table 1). The intersected DEGs in GSE121248 are shown in Figure 1(c).

### 3.2. GO and KEGG Pathway Enrichment Analyses

**Figure 2:**
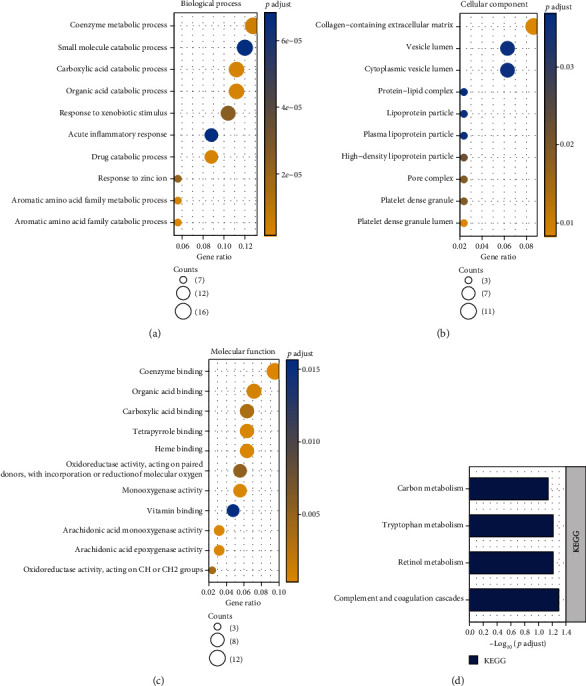
GO annotation and KEGG pathway enrichment analysis: (a) the biological processes; (b) the cellular components; (c) the molecular functions; (d) the KEGG pathway enrichment analysis of DEGs.

The Enrichr database was used for GO and KEGG enrichment analysis to explore the biological role of the screened DEGs.  Figure 2 lists the top 10 enriched GO and KEGG pathways. GO annotation revealed that significantly enriched biological processes associated with the 145 DEGs included coenzyme metabolic process, small molecule catabolic process, carboxylic acid, organic acid catabolic process, etc. (Figure 2(a)). The top four significantly enriched terms in cell component analysis were collagen-containing extracellular matrix, vesicle lumen, cytoplasmic vesicle lumen, and protein-lipid complex (Figure 2(b)). Moreover, the top four significantly enriched molecular function terms included coenzyme binding, organic acid binding, carboxylic acid, and tetrapyrrole binding (Figure 2(c)). Finally, the top four significantly enriched signaling pathways for the 145 DEGs were carbon metabolism, tryptophan metabolism, retinol metabolism, and complement and coagulation cascades (Figure 2(d)).

### 3.3. MAD2L1 Is a Core Gene in the PPI Network

**Figure 3:**
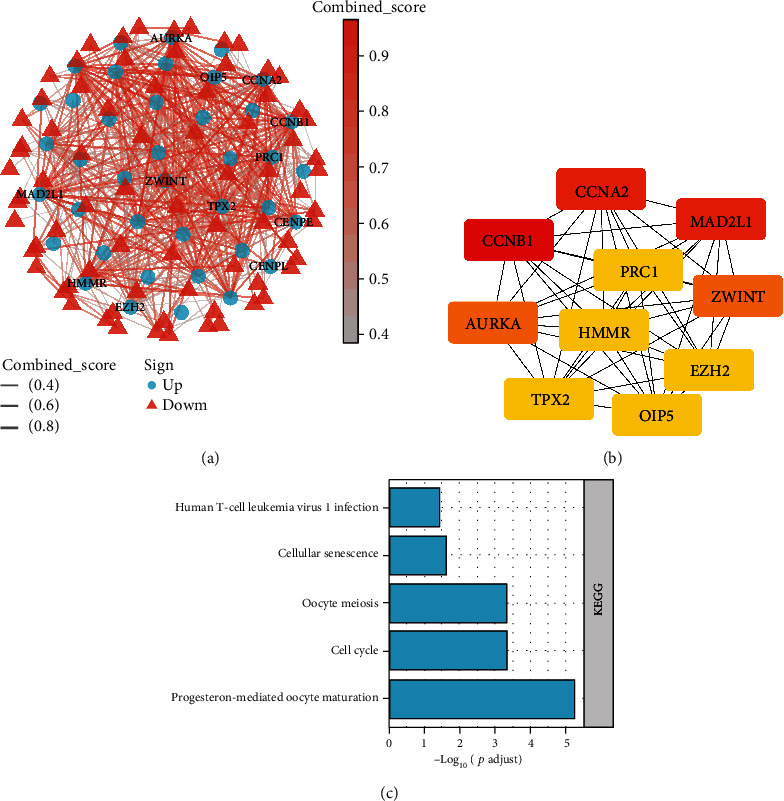
The PPI network and hub gene identification: (a) PPI network of 145 DEGs; (b) PPI network of the top 10 hub genes; (c) KEGG enrichment analysis of the 10 hub genes.

The STRING database was employed to construct the PPI network of these 145 DEGs. 145 nodes (genes) and 484 edges (interactions) were observed in the constructed PPI network (Figure 3(a)). We eventually selected the top 10 hub genes by their connectivity level, including “CCNB1” (score = 29), followed by “MAD2L1” (score = 27), “CCNA2” (score = 27), “AURKA” (score = 26), “ZWINT” (score = 25), “TPX2” (score = 25), “EZH2” (score = 25), and “HMMR” (score = 25) (Supplementary Table 2). The interactions among these ten hub genes were further visualized (Figure 3(b)). KEGG analysis revealed that the significantly enriched pathways for the ten hub genes were progesterone-mediated oocyte maturation, cell cycle, oocyte meiosis, cellular senescence, and human T-cell leukemia virus one infection. Based on the PPI network, we identified CCNB1, MAD2L1, and CCNA2 as the top genes with higher connectivity degrees with other genes, suggesting their core position in the network. Given that CCNB1 has been widely reported in various tumors, we selected MAD2L1 as the study candidate for our follow-up work.

### 3.4. Expression and Survival Analysis of MAD2L1

**Figure 4:**
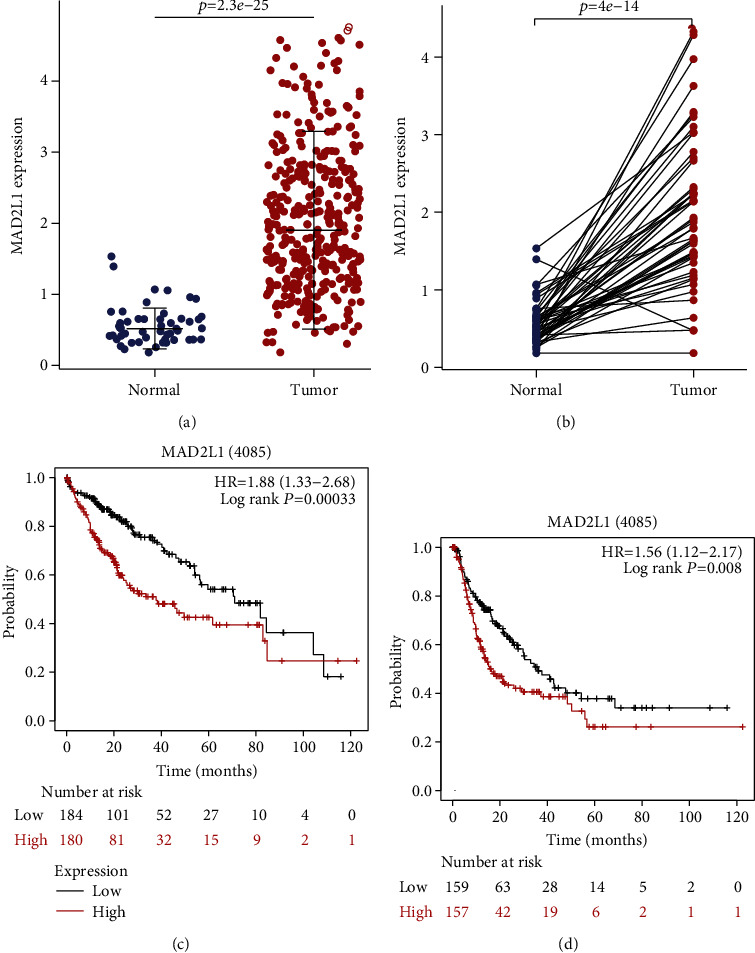
Expression and survival analysis of MAD2L1: (a) the mRNA levels of MAD2L1 in TCGA in HCC tissues and adjacent liver tissues; (b) the mRNA levels of MAD2L1 in paired HCC and adjacent liver tissues in TCGA database; (c) Kaplan-Meier survival analysis for the overall survival of MAD2L1 in HCC; (d) Kaplan-Meier survival analysis for the recurrence-free survival of MAD2L1 in HCC.

The mRNA expression level of MAD2L1 in HCC was further assessed in TCGA. We found that MAD2L1 expression was significantly upregulated in HCC tissues than in paracancerous tissues (Figures 4(a) and 4(b)). Additionally, the prognostic value of MAD2L1 expression in HCC was assessed by the Kaplan-Meier plotter. Patients with high expression of MAD2L1 had a shorter survival time (Figures 4(c) and 4(d)). Overall, these results suggest that MAD2L1 expression is higher in HCC tissue than in adjacent liver tissue, and MAD2L1 is an adverse prognostic factor.

### 3.5. GSEA for MAD2L1-Associated Signaling Pathways in HCC

**Figure 5:**
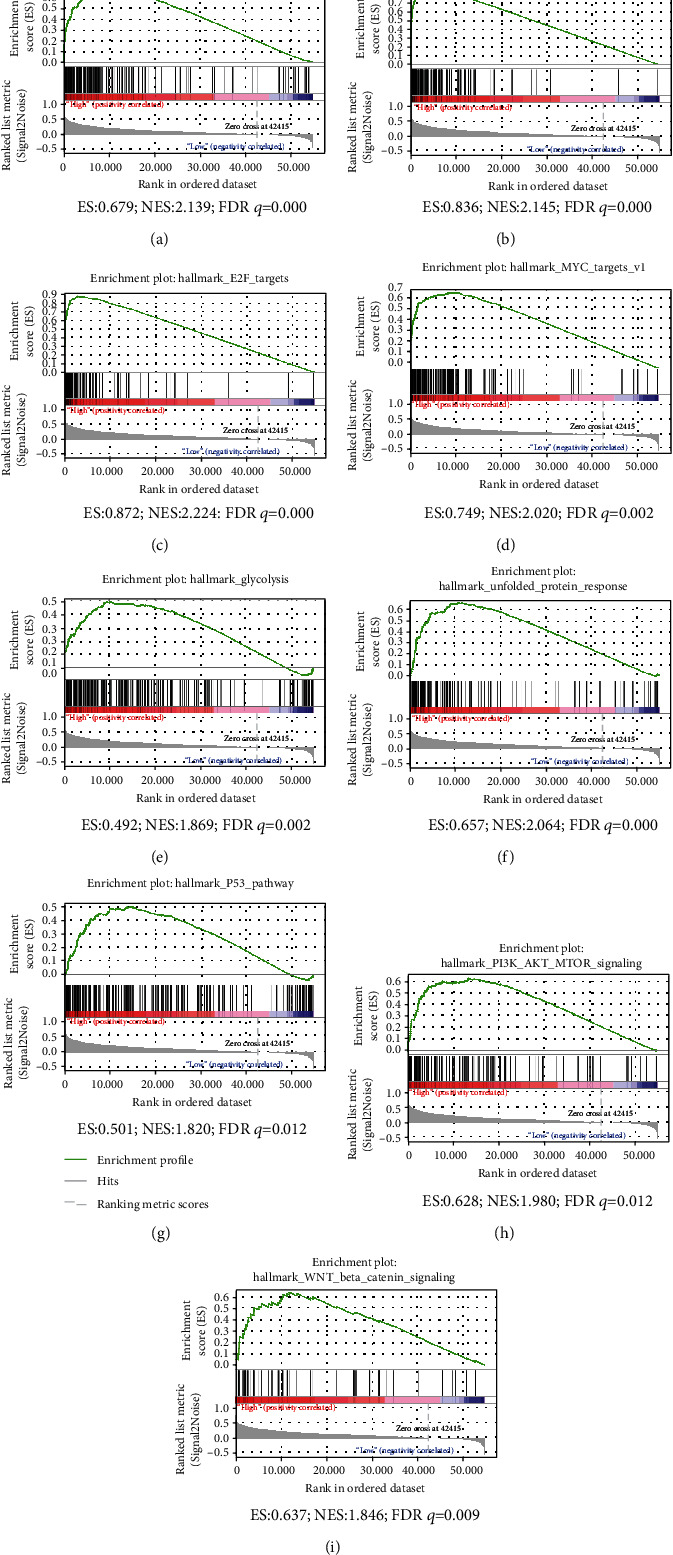
GSEA pathways enriched in samples with high MAD2L1 expression: (a) DNA repair; (b) G2M checkpoint; (c) E2F targets; (d) MYC targets; (e) glycolysis; (d) unfold protein response; (g) P53 pathway; (h) PI3K/AKT/TOR pathway; (i) wnt-*β-*catenin pathway. ES: enrichment score; NES: normalized enrichment score; FDR: false discovery rate.

To explore the potential biological functions of MAD2L1, we performed GSEA for samples with low and high MAD2L1 expression to predict MAD2L1-related signaling pathways. The significantly enriched terms upregulated in the high MAD2L1 group were “DNA repair,” “G2M checkpoint,” “E2F targets,” “MYC targets,” “glycolysis,” “unfolded protein response,” “P53 signaling pathway,” “PI3K/AKT/mTOR signaling pathway,” and “Wnt/*β*-catenin signaling pathway” (Figure 5).

### 3.6. High Expression of MAD2L1 in HCC

**Figure 6:**
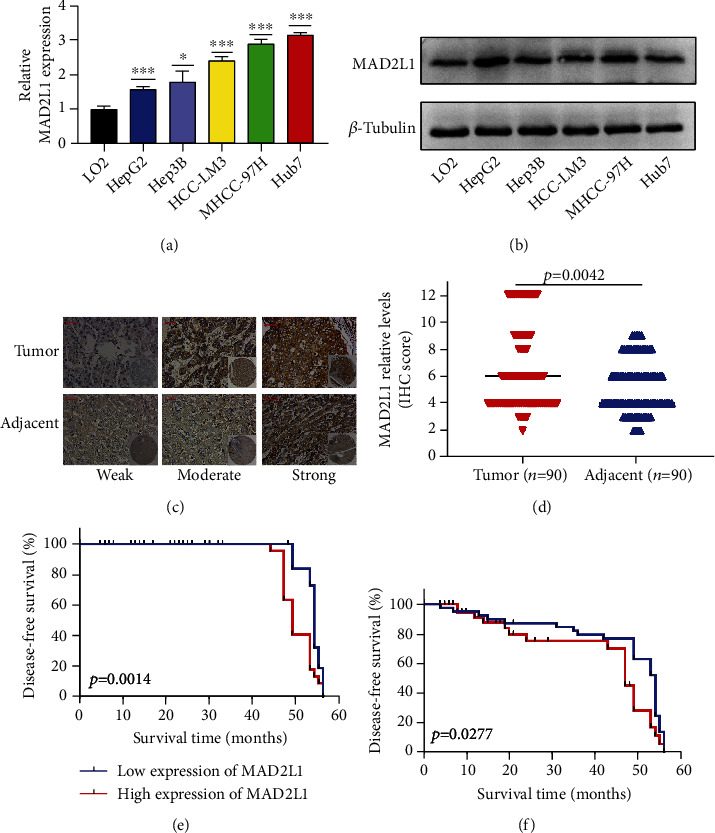
High expression of MAD2L1 in HCC: (a) the expression levels of MAD2L1 were analyzed by qRT-PCR in HCC cells and normal hepatocytes; (b) The expression levels of MAD2L1 were analyzed by western blot in HCC cells and normal hepatocytes; (c) MAD2L1 expression in tumors and adjacent samples was detected by IHC on tissue arrays (scale bar: 50 *μ*m); (d) expression of MAD2L1 was scored by IHC on tissue arrays in tumor and adjacent samples; (e) Kaplan-Meier survival analysis for disease-free survival; (f) Kaplan-Meier survival analysis for disease-free survival.

We selected five HCC and one hepatocyte cell line to evaluate the expression of MAD2L1 using real-time quantitative PCR and western blot. The RNA and protein levels of MAD2L1 were upregulated (Figures 6(a) and 6(b)). To further determine the significance of MAD2L1 expression, IHC staining was performed in a cohort comprising 90 cases of primary HCC paired with noncancerous tissue (Figures 6(c) and 6(d)). Survival analysis showed that overexpression of MAD2L1 was associated with poor prognosis, reducing the overall survival time (*P* = 0.014) and disease-free survival time (*P* = 0.028) of HCC patients (Figures 6(e) and 6(f)).

A correlation was found between MAD2L1 expression and the clinicopathological features of HCC. High expression of MAD2L1 was linked with Edmondson-Steiner grading (*P* = 0.019) and tumor size (*P* = 0.042). During the univariate analysis, Edmondson-Steiner grading (*P* < 0.001), GGT (*P* = 0.035), and AJCC (*P* = 0.021) were significantly correlated (Table 1). To determine whether MAD2L1 was a prognostic factor independent of HCC, we conducted a multifactorial Cox regression analysis based on the expression level of MAD2L1 adjusted for the Edmondson-Steiner grade, GGT, and AJCC of HCC patients. Importantly, we found that MAD2L1 expression and Edmondson-Steiner grading were independent prognostic factors for HCC (Table 2).

## 4. Discussion

Despite recent advances in diagnosis and treatment, hepatocellular carcinoma remains one of the most lethal cancers globally [23–25]. Although the treatment landscape continues to be challenging given the heterogeneity of tumors and the evolutionary nature of cancer, molecular pathology offers much promise for HCC in terms of molecular diagnosis and targeted therapy [26–28]. Indeed, in the current era of precision medicine, it is of great benefit to explore abnormal molecular genetic alterations in tumors through various bioinformatics analysis tools to predict the survival prognosis of tumor patients.

The GEO database is one of the most common public databases used by researchers worldwide to explore genetic abnormalities in various cancers [29–32]. In this study, we first selected four different cDNA expression profiles, GSE121248, GSE101685, GSE85598, and GSE62232, from the GEO database to analyze the DEGs in HCC compared with normal liver tissues and screened 145 genes (including 42 upregulated and 153 downregulated genes). Interestingly, although these DEGs were enriched in different cellular locations, most of the up- and downregulated DEGs were involved in biological processes related to metabolism and energy regulation.

To narrow down the number of “candidate” DEGs and determine potential “key” genes for HCC development [30, 31, 33, 34], a PPI network of 145 DEGs was constructed to visualize the relationships between genes, followed by functional analysis. Based on their connectivity level, the top ten hub genes identified included CCNB1, MAD2L1, CCNA2, AURKA, ZWINT, HMMR, TPX2, EZH2, and OIP5. Most importantly, MAD2L1 was the highest-ranked gene among these ten genes in the PPI network.

In previous studies, the differential expression of MAD2L1 in many tumors was analyzed using the TIMER2.0 database [35, 36]. It was found that the expression of the MAD2L1 gene was higher in BLCA, BRCA, CESC, CHOL, COAD, ESCA, GBM, HNSC, KIRC, LUAD, LUSC, PRAD, READ, STAD, and UCEC than in their corresponding paracancerous tissues. There are currently no reports of MAD2L1 expression in HCC and its potential prognostic impact in the literature. In a study by Wei et al., bioinformatics analysis was used to demonstrate that NDC80 and MAD2L1 were potential biomarkers for the diagnosis of non-small-cell lung cancer [37]. Moreover, CDK1 and MAD2L1 were reported by Lu et al. as prognostic markers in rhabdomyosarcoma [38]. To the best of our knowledge, this is the first comprehensive study to assess the expression of MAD2L1 in HCC using TCGA database [39]. We provided compelling evidence that MAD2L1 gene expression levels are significantly higher in HCC patients than in adjacent paraneoplastic tissues, as confirmed by IHC. Kaplan-Meier survival analysis showed high expression of MAD2L1 in HCC correlated with shorter OS and DFS. This finding was also validated in other datasets. Multivariate Cox analysis further confirmed that high expression of MAD2L1 was an independent risk factor for OS in patients with HCC. Other clinicopathologic features, including Edmondson-Steiner grade and tumor size, were also associated with a worse prognosis in HCC.

Herein, GSEA results showed that MAD2L1 was associated with DNA repair, G2M checkpoint, p53 signaling pathway, PI3K/AKT/mTOR signaling pathway, and Wnt/*β*-catenin signaling pathway in cancer. It is widely acknowledged that DNA replication ensures that cellular genetic information is accurately copied and correctly transmitted to offspring cells [32, 40, 41]. However, DNA replication is prone to interference and damage under various pressures in the body, leading to stagnant DNA replication, affecting genome stability, and even inducing apoptosis [42], necrosis [43], and carcinogenesis [44]. Pathway enrichment analysis suggested that MAD2L1 affected the pathogenesis of proliferation and apoptosis in hepatocellular carcinoma via the above pathways. MAD2L1 has been documented to be associated with female breast cancer [45], where it is usually deleted or amplified simultaneously with BUB1B. Therefore, these two genes are commonly tested in ductal breast carcinoma patients to aid clinicians in selecting anticancer agents [46].

Furthermore, MAD2L1 has been reported in glioblastoma as a target of tumor suppressors, including miR-30a-3p, which inhibited the proliferation of gastric cancer cells [47]. In addition, the cell cycles were arrested at the G0/G1 phase.

To the best of our knowledge, no evidence of an association between HCC and genetic abnormalities involving MAD2L1 has been reported. Although our approach can provide new insights into the correlation between MAD2L1 and HCC, certain limitations were noted in this study. First of all, only GEO and TCGA datasets were analyzed, which may be a source of sample bias. To increase the robustness of our findings and ensure their implementation at the clinical level, the sample size should be further expanded, with additional clinical factors included in future studies. Finally, experimental verification is required to elucidate the mechanism of MAD2L1 in HCC development in vitro and in vivo. In summary, our study provided significant insights into better understanding the pathogenesis of HCC; however, our findings were not robust enough to classify MAD2L1 proteins as new potential drug targets in HCC. In addition, many questions remain to be addressed. The specific mechanism of MAD2L1 in HCC remains unknown, nor is it clear whether MAD2L1 is associated with chemoresistance in HCC. Accordingly, further research on the mechanisms at the molecular level is required to improve the clinical treatment of this patient population.

## 5. Conclusions

In short, we identified 145 DEGs in HCC based on the GEO database, and the gene MAD2L1 was found to be a core component of the PPI network of DEGs. The analysis of online databases and IHC, qPCR, and WB assays demonstrated abnormal overexpression of MAD2L1 in HCC compared to paraneoplastic tissues. Survival analysis suggested that high MAD2L1 expression was correlated with a poor prognosis. In addition, the biological processes and signaling pathways associated with MAD2L1 were preliminarily explored. Further investigations are essential to improve our understanding of the clinical applications they may hold.

## Figures and Tables

**Table 1:** Correlations between MAD2L1 expression and the clinicopathological features of hepatocellular carcinoma patients.

Characteristics	*n*	MAD2L1 expression		*P* value
		Low	High	
Age (years)				
>50	50	20 (40.00%)	30 (60.00%)	0.343
≤50	40	20 (50.00%)	20 (50.00%)	
Gender				
Male	80	34 (42.50%)	46 (57.5%)	0.476
Female	10	6 (60.00%)	4 (40.00%)	
AJCC stage				
I	63	31 (49.21%)	32 (50.79%)	0.165
II-III	27	9 (33.33%)	18 (66.67%)	
HBsAg				
Negative	19	10 (52.63%)	9 (47.37%)	0.419
Positive	71	30 (42.25%)	41 (57.75%)	
AFP (*μ*g/L)				
>400	33	15 (45.45%)	18 (54.54%)	0.883
≤400	57	25 (43.86%)	32 (56.14%)	
Total bilirubin (*μ*mol/L)				
>20	15	7 (46.67%)	8 (53.33%)	0.850
≤20	75	33 (44.00%)	42 (56.00%)	
ALT (U/L)				
>45	32	15 (46.88%)	17 (53.12%)	0.730
≤45	58	25 (43.30%)	33 (56.70%)	
GGT (U/L)				
>40	59	24 (40.68%)	35 (59.32%)	0.321
≤40	31	16 (51.61%)	15 (48.39%)	
Edmondson-Steiner grade				
I & II	53	29 (54.72%)	24 (40.68%)	0.019
III & IV	37	11 (29.73%)	26 (70.27%)	
Tumor number				
1	79	35 (44.30%)	44 (55.70%)	0.943
>1	11	5 (45.45%)	6 (54.55%)	
Tumor size (cm)				
>5	28	8 (28.57%)	20 (71.43%)	0.042
≤5	62	32 (51.61%)	30 (48.39%)	

**Table 2:** Univariate and multivariate survival analyses.

Characteristics	OS					
	Univariate			Multivariate		
	HR	95% CI	*P* value	HR	95% CI	*P* value
MAD2L1expression	11.511	(3.471-38.110)	<0.001	8.644	(2.543-29.382)	0.001
Low vs. high						
Age (years)	0.721	(0.352-1.476)	0.371			
≤50 vs. >50						
Gender	0.520	(0.124-2.176)	0.371			
Male vs. female						
AJCC stage	2.262	(1.128-4.536)	0.021	1.230	(0.599-2.527)	0.512
I vs. II-III						
HBsAg	1.001	(0.429-2.336)	0.998			
Negative vs. positive						
AFP (ng/mL)	0.888	(0.434-1.818)	0.746			
≤400 vs. >400						
Total bilirubin (*μ*mol/L)	1.113	(0.428-2.893)	0.826			
≤20 vs. >20						
ALT (U/L)	0.935	(0.457-1.912)	0.853			
≤45 vs. >45						
GGT (U/L)	0.386	(0.159-0.937)	0.035	0.572	(0.231-1.416)	0.227
≤40 vs. >40						
Edmondson-Steiner grade	5.198	(2.384-11.336)	<0.001	3.640	(1.589-8.333)	0.002
I-II vs. III-IV						
Tumor number	1.810	(0.742-4.417)	0.192			
Single vs. multiple						
Tumor size (cm)	0.504	(0.250-1.018)	0.056			
≤5 vs. >5						

## Data Availability

The data used to support the findings of this study are available from the corresponding author upon request.

## Supplementary Materials

Supplementary 1 Supplementary Table 1: the common DEGs of the four GEO datasets (adj.*P*val.<0.05, ∣logFC | >1.0).

Supplementary 2 Supplementary Table 2: the top ten hub genes in the PPI network.
