# Supplementary material for: Identification of MAD2L1 as a Potential Biomarker in Hepatocellular Carcinoma via Comprehensive Bioinformatics Analysis
Source: Biomed Res Int. 2022 Jan 28;2022:9868022. doi: 10.1155/2022/9868022 (PMC8817109; doi:10.1155/2022/9868022)
Supplement: Supplementary 1 — Supplementary Table 1: the common DEGs of the four GEO datasets (adj.Pval.<0.05, ∣logFC | >1.0). [file 9868022.f1.pdf]

Supplementary Table 1. **The common DEGs of four gene expression profiles (adj. Pval. <0.05, |logFC|>1.0).**

Common DEGs	Gene symbol
Upregulated DEGs	ZWINT; UHRF1; UBE2T; UBE2C; TRIP13; TPX2; TP53I3; TKT; STMN1; STIL; SRXN1; SQLE; SPINK1; SMYD3; SLC38A6; RFC4; RAD51AP1; PTTG1; PRC1; PLVAP; OIP5; NUSAP1; MELK; MDK; MCM4; MAD2L1; ITGA6; HMMR; EZH2; ESM1; ECT2; CENPL; CENPE;CDKN2B; CCNB1;CCNA2; CCDC34; CAP2; AURKA; ATAD2; ASPM; AKR1B10
Downregulated DEGs	ZGPAT; XDH; VIPR1; TUBE1; TMEM27; TDO2; TAT; STEAP3;SPP2; SOCS2; SLC7A2; SLC22A1; SKAP1; SIGIRR; SHBG; SDS;S100A8; RNF165; RND3; RDH16; RCL1; RCAN1; PPARGC1A; PLAC8; PGLYRP2; PCK1; PCDH9; PANK1; PALM2; OGDHL; NR4A2; NR1I2; NNMT; MYO10; MT1H; MT1G; MT1F; MS4A6A; MASP1; MARCO;LYVE1; LY6E; LIFR;LCAT; KMO; KLKB1; KCNMA1; ITIH4; ITGA9; IL33; IL1RAP; IGFBP3; IGFALS; IDO2; ID1; HSD17B2; HBB; HAO1; HAMP; GSTZ1; GSPT2; GPR128; GHR; GCKR; GCH1; GBA3; GADD45B; FTCD; FOSB; FOS; FEZ1; FCN3; FBP1;FAM149A; F9; ECM1; DNASE1L3; CYP4V2; CYP4A11; CYP2C9; CYP2C8;CYP2C18; CXCL2; CXCL12; COLEC11; CFP; CETP; CDA; CD1D; CA2;C8B; C8A; C6; BGN; BASP1; ASS1; APOA5; ANXA10; ANGPTL6; AFM;ACSL1; ACADS; AADAT
