# Supplementary material for: Identification of MAD2L1 as a Potential Biomarker in Hepatocellular Carcinoma via Comprehensive Bioinformatics Analysis
Source: Biomed Res Int. 2022 Jan 28;2022:9868022. doi: 10.1155/2022/9868022 (PMC8817109; doi:10.1155/2022/9868022)
Supplement: Supplementary 2 — Supplementary Table 2: the top ten hub genes in the PPI network. [file 9868022.f2.pdf]

Supplementary Table 2Top ten hub genes with higher degree of connectivity.

Gene symbol	Gene description	Score
CCNB1	Cyclin B1	29
MAD2L1	mitotic arrest deficient 2 like 1	27
CCNA2	Cyclin B1	27
AURKA	aurora kinase A	26
ZWITN	ZW10 interacting kinetochore protein	26
PRC1	protein regulator of cytokinesis 1	25
HMMR	hyaluronan mediated motility receptor	25
TPX2	TPX2 microtubule nucleation factor	25
EZH2	enhancer of zeste 2 polycomb repressive complex 2 subunit	25
OIP5O	Opa interacting protein 5	25
